# Variations in the Surface Integrity of Ti-6Al-4V by Combinations of Additive and Subtractive Manufacturing Processes

**DOI:** 10.3390/ma13081825

**Published:** 2020-04-13

**Authors:** Roland Bejjani, Erik Bamford, Stefan Cedergren, Andreas Archenti, Amir Rashid

**Affiliations:** 1 Department of Mechanical Engineering, Lebanese American University, Byblos 36, Lebanon; 2 Department of Production Engineering, Manufacturing and Metrology Systems, KTH, Royal Institute of Technology, 100 44 Stockholm, Sweden; erik.bamford@hotmail.com (E.B.); sced@kth.se (S.C.); archenti@kth.se (A.A.); amirr@kth.se (A.R.)

**Keywords:** additive manufacturing, titanium, machining, surface integrity

## Abstract

Additive manufacturing (AM) has recently been accorded considerable interest by manufacturers. Many manufacturing industries, amongst others in the aerospace sector, are already using AM parts or are investing in such manufacturing methods. Important material properties, such as microstructures, residual stress, and surface topography, can be affected by AM processes. In addition, a subtractive manufacturing (SM) process, such as machining, is required for finishing certain parts when accurate tolerances are required. This finish machining will subsequently affect the surface integrity and topography of the material. In this research work, we focused on the surface integrity of Ti-6Al-4V parts manufactured using three different types of AM and finished using an SM step. The aim of this study was to gain an understanding on how each process affects the resulting surface integrity of the material. It was found that each AM process affects the materials’ properties differently and that clear differences exist compared to a reference material manufactured using conventional methods. The newly generated surface was investigated after the SM step and each combination of AM/SM resulted in differences in surface integrity. It was found that different AM processes result in different microstructures which in turn affect surface integrity after the SM process.

## 1. Introduction

Additive manufacturing (AM) is an increasingly popular manufacturing method in the aerospace and biomedical sectors, amongst others. Frazier [1] reviewed many of the existing 3D printing techniques and commented that “AM has the potential to revolutionize the global parts manufacturing and logistics landscape”. While AM can produce near net shape parts, an additional conventional subtractive manufacturing step, such as machining, is specifically required when high dimensional tolerances and a defined surface finish are required. For example, a smooth surface on turbine blades in airplanes is important for better fatigue performance and less corrosion [2].

While different AM techniques are available today, the challenge is to control the input parameters, such as powder types, energy type and feeding methods, and to be able to predict quality output parameters.

Direct energy deposition (DED) and powder bed fusion (PBF) are common AM methods used for processing titanium and its alloys [3], but new 3D printing techniques are constantly in development [4]. There are different ways to build 3D printed metal designs, but it is important to understand the resulting material properties for their proper implementation.

Materials made using different 3D printing methods exhibit different microstructures and mechanical properties which alter their hardness and machinability. The rapid temperature changes that occur during 3D printing cause radical changes in the microstructure which in turn results in variations in the hardness of the material [5].

Different material properties may be required depending on the application of the manufactured part, and choosing which AM method is more appropriate when later combined with finish machining can be a challenge. It is thus of great relevance to compare different AM techniques and learn more about how later machining operations will affect the material properties and surface integrity. Shoulder milling is a common machining operation technique used to remove the rough surface finish resulting from 3D printing leading to better controlled surface tolerance and roughness.

### 1.1. Different AM Techniques and Their Properties

Selective laser melting (SLM) is a particular PBF method, where the laser beam fuses and melts a powder bed thus building layers of material [6]. The laser beam raises the temperature of the powder to form what is called a melt pool. After the heating cycle, the material rapidly cools down and solidifies. The subsequent cross-section scanned by the laser is indexed at a lower level, and a new layer is deposited. Due to the high cooling rates that occur as a result of the requirement to maximize part density, the microstructure of as-built SLM-fabricated Ti-6Al-4V components is composed of acicular α’ martensite [7,8]. Consequently, SLM-processed Ti-6Al-4V components tend to have high tensile strength but poor ductility [9]. Fabrication of Ti-6Al-4V using this method has been shown to yield similar or better material properties compared to forged Ti-6Al-4V in terms of tensile and yield strength. Values above 1 GPa have been reported [9]. It must be noted that various heat treatments of SLM Ti-6Al-4V can significantly increase elongation at break [10].

**Figure 1:**
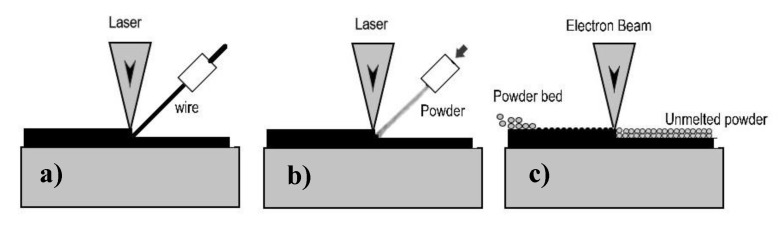
Schematic representation of metal additive manufacturing (AM) processes used: (**a**) laser wire melting with wire feeding system; (**b**) laser metal deposition system; (**c**) powder bed fusion with electron beam melting.

Electron beam melting (EBM), which was first developed in Sweden [11], is another PBF process which uses an electron beam instead of the typical laser beam to melt the powder. Direct energy deposition (DED) also completely melts the powder. This method shares similarities with welding and is known to affect the material’s properties [12]. Sandia National Research Lab integrated a laser beam into the DED in a method later called LENS (laser engineered net shaping) [13]. Laser wire (LW) melting deposition is also a DED method. In this case, the laser beam provides the thermal energy to heat the wire which is used to build the printed part. Figure 1 shows the three AM methods used in the present work.

### 1.2. Material Properties in AM

#### 1.2.1. Parameters Affecting AM Material Properties

- Heating and cooling rates

All AM techniques include a heating followed by a cooling cycle which will play a role in the final build of the Ti-6Al-4V microstructures and phases. For example, if an α’ martensite microstructure is formed, the microstructure of the following build layers will be highly affected by the stability of this previously formed martensite [14].

After the heating cycle, the temperature reaches the β transus temperature and the α phase starts to form during cooling, a process which is initiated at the prior β grain boundaries. With a further decrease in temperature, the α phase grows into the β grains, either in a platelet form or along the β grain boundaries, depending on the cooling rate. The cooling rates also affect the size and distribution of α laths inside the prior β grains [15,16,17]. The thickness of α laths decreases if the cooling rates are increased. Hence, regulating the cooling rate during solidification is the most common method used to control the microstructure [18].

- Process parameters

The AM process parameters are linked to a rise in temperature, differences in temperature gradients, and changes in cooling rates therefore affecting material properties such as microstructures, phases, and internal stresses. Often these process parameters are chosen in a way to reduce the material porosity; however, this will directly affect all aforementioned properties [19].

- Metal powders

It is still not well understood how the metal powders used in AM influence the resulting microstructures, and a limited number of studies have focused on the characterization of powders in terms of their effect on microstructure, phases, and morphology [20]. Note that all mentioned AM processes, with the exception of wire-based processes which do not employ any powder, involve repeated full melting of the powder.

#### 1.2.2. Properties Affected by AM Parameters

- Microstructures

Material microstructures are greatly affected by the type of AM process. For example, during a laser metal deposition (LMD) process, the material can be held at a high temperature for a longer time using a high energy laser which allows the grains to coarsen and typically adopt an equiaxed nature [13]. In contrast, the use of a low energy laser with a fast scanning speed will produce thinner molten pools with thin columnar grains [21].

Stored energy plays an important role in the recrystallization process, and its contributions can be categorized as elastic energy and dislocation density. Elastic energy relates to residual stress, which is characterized by grain-to-grain interactions, while dislocation density is mainly confined inside the grains [22].

For SLM, the bulk energy density E was shown to be associated with the formation of fine acicular martensite and with the lamellar α phase [23]. Partial melting of the pool and a more uniform temperature inside the molten pool will occur if the energy density E is too low (less than 533 J/mm^3^), leading to a more equiaxed microstructure. An increase of the bulk energy will lead to the destruction of the prior β grain boundaries and to the formation of a Widmanstätten structure. With a higher energy density (600–1333 J/mm^3^), a higher temperature gradient in the melt will result in a fine acicular martensitic structure.

- Residual stress

The thermal energy plays a major role in maximizing part density and is associated to an increase in the temperature gradient, followed by rapid cooling. This cycle of thermal energy and cooling will affect the internal stresses frequently observed in Ti-6Al-4V AM components.

It was found that the stored energy in many AM materials is mainly due to the dislocation density, as the stored energy in the form of residual stress is not on a high enough level to induce recrystallization. Martensitic transformations are sometimes considered as a method to increase the dislocation density inside the material [22]. Lath martensites are typically associated with high dislocation densities, as they can accommodate high strains due to the diffusionless martensitic transformation [24]. Therefore, a higher fraction of martensite is accompanied by high dislocation densities [25].

### 1.3. Machining Titanium Alloys

Many variables are involved in the machining of metals. Workpiece conditions (e.g., chemical composition, hardness, microstructure), physical properties (e.g., thermal softening, thermal conductivity, work hardening), and cutting conditions (e.g., cutting parameters, tool geometry, tool material) can act in combination or independently to affect the machinability of the workpiece material [26]. Thermal softening is a process where the material loses its strength at elevated temperatures, while work hardening (i.e., strain hardening) is the hardening process that occurs in the machined material when it is plastically deformed [27]. Thermal softening and work hardening operate simultaneously in the workpiece material, and it has been reported that work hardening reduces the thermal softening effect [28]. 

Titanium is a hard-to-cut material due to its low thermal conductivity and modulus of elasticity, and to its high chemical reactivity and temperature strength [29,30,31]. Low thermal conductivity results in high temperatures at the tool–chip interface, since the thermal energy does not dissipate easily by conduction. Titanium alloys are chemically stable at low temperatures, but they easily react with cutting tool materials, leading to diffusion wear if the temperature surpasses 500 °C [30].

Previous studies have investigated surfaces made by one specific method of AM before [32] and after [33,34] machining. However, these preliminary studies were performed only for turning and with one type of AM material. No research paper comparing the surface integrity of titanium alloy parts made by different AM techniques in combination with SM could be found in the literature.

In this work, we focused on surface integrity when machining similarly alloyed Ti-6Al-4V workpieces manufactured using different AM techniques. The sample materials used were made using blown powder laser metal deposition (LMD), laser wire metal deposition (LW), and powder bed fusion by electron beam melting (EBM) (see Figure 1). A reference forged material was also included. Equal-sized pieces were subjected to shoulder milling. Since each AM process produces different surface qualities (including topographies), which may later affect the subsequent subtractive machining forces, the samples’ surfaces were all cleaned by machining to remove the superficial surface layer.

In this work, AM processes applied for ready-to-use industrial applications were employed. As for the SM, finishing shoulder milling parameters were used.

As described in the previous sections, each AM process involves many control parameters and can result in different material properties. It will be impossible to compare all combinations of AM methods and SM machining parameters. 

Instead, in this work we used AM materials produced without any altered process parameters, in combination with a commonly used SM finishing process. The main objective of this investigation was the understanding of the surface integrity resulting from the combination of different AM and SM processes. This was done by analyzing the cutting forces, chip formation, and microstructure as well as the surface integrity in terms of residual stresses, strained surface layer, micro-hardness, and surface roughness.

No previous studies could be found in the literature concerning the effect of a material’s texture orientation on milling. However, since previous work on turning Al showed an effect of texture direction on cutting forces [35], we expected a similar behavior when milling different AM Ti-6Al-4V with varying microstructures and textures. In this work, we used a novel approach to relate the effect of grain texture on milling machinability and its relations to cutting forces.

## 2. Experimental Setup

The research work consisted in machining three types of Ti-6Al-4V AM materials and a reference material manufactured by a traditional method. The objectives of this work were to identify differences in surface integrity after milling SM and to investigate the machinability of the different AM materials.

All material samples were polished, and the microstructures were analyzed. The cutting forces were measured and compared to verify how they varied when machining different AM materials. After milling, the surface integrity of the generated surface was analyzed for surface hardness, surface strain, residual stress, and surface topography at different scales.

**Figure 2:**
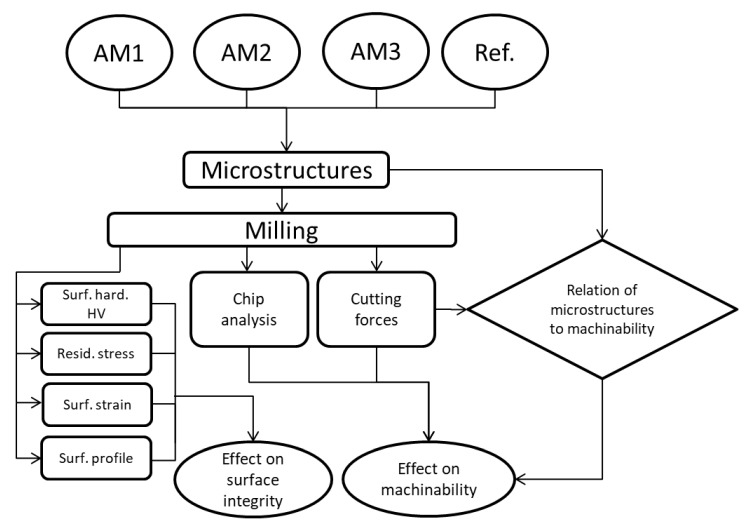
Flowchart showing the experimentation process. (AM, Additive Manufacturing; Ref., Reference; Surf., Surface; hard., hardness; Resid., Residual).

Since chip formation analysis can help understand the material’s cutting behavior and machinability, chips were collected during milling, and samples were polished and analyzed under the microscope. Figure 2 shows the experimental workflow. To help understand how different microstructures can affect the machinability, a relation between the cut surface and interception with the microstructure grains was proposed. 

The cutting tools were supplied by Sandvik Coromant as specified in Table 1.

**Figure 3:**
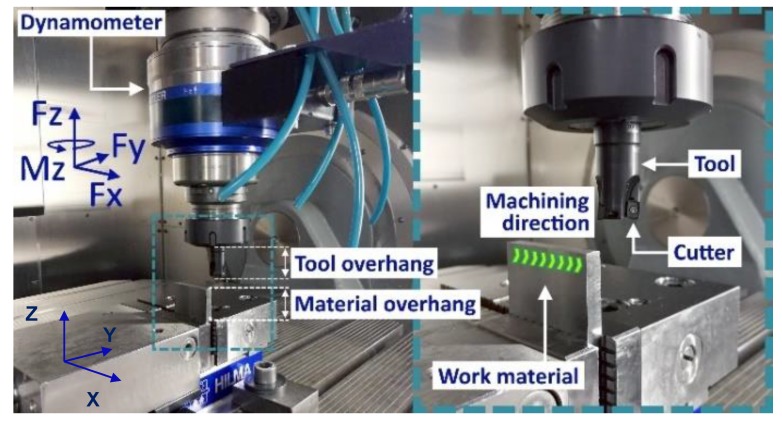
Machining setup and force directions.

The milling tests were performed in a Hermle C50 machining center. Force measurements were conducted with a Kistler rotating cutting dynamometer (RCD) of type 9171A (Zürich, Switzerland). The machining setup is shown in Figure 3. The measurements were made for the force along the feed direction (Fx), the force perpendicular to the feed direction (Fy), the passive force (Fz) along the spindle, and the spindle moment (Mz).

The AM samples were produced as ready-to-use by an aerospace manufacturer using their own standard parameters. All AM machines were purpose-built or modified—as was the case for EBM using ARCAM as the base—by the aerospace manufacturer.

No heat treatment was performed, as it would have added more complexity to the surface integrity analysis after SM, since subjecting AM parts to heat treatment may result in different material properties and, therefore, different machinability. A typical requirement for aerospace manufacturers is meeting the specifications for surface integrity after SM. If these requirements can be met without a heat treatment, this reduces the complexity of the complete process to obtain a final product.

The dimensions of the work material samples were 40 × 70 × 7 mm. The material overhang was 30 mm, and the tool overhang 32 mm. Some AM parts are considered ready for usage, but as is the case for dimensional accurate parts, finish machining is typically performed. The cutting parameters are shown in Table 2.

**Figure 4:**
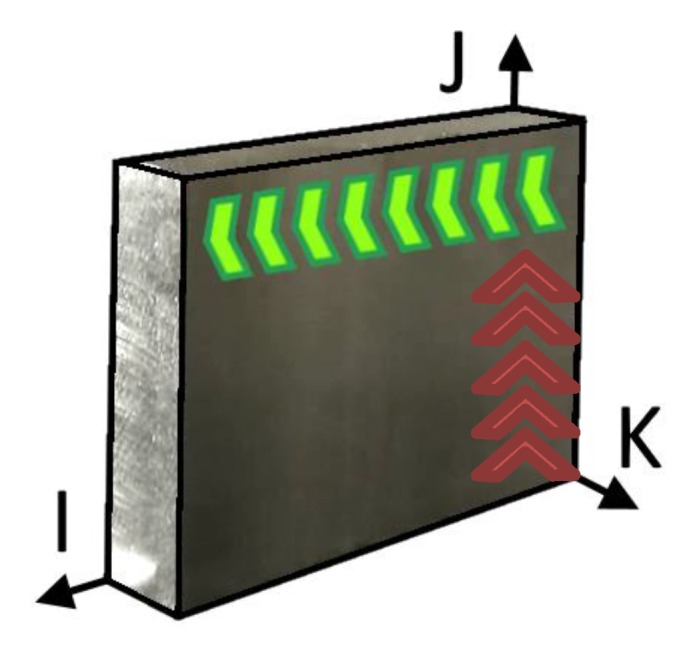
Schematic overview of the two machining directions in the IJ-plane of the workpiece. The green arrows show the machining parallel to the I-direction, and the red arrows show the machining parallel to the J-direction.

The work materials were machined in two different directions in the IJ-plane: parallel to I and parallel to J, according to the green and red arrows in Figure 4. Machining along the K-direction was not performed due to the short material length.

Table 3 displays the build direction, in terms of plane deposition, for each AM material. Due to the nature of the AM machines, the EBM material was built along the thinner side, so perpendicular to the IJ direction. Later on, shoulder milling was performed on the thinner side of the samples as would be done for a part suitable for later use.

**Figure 5:**
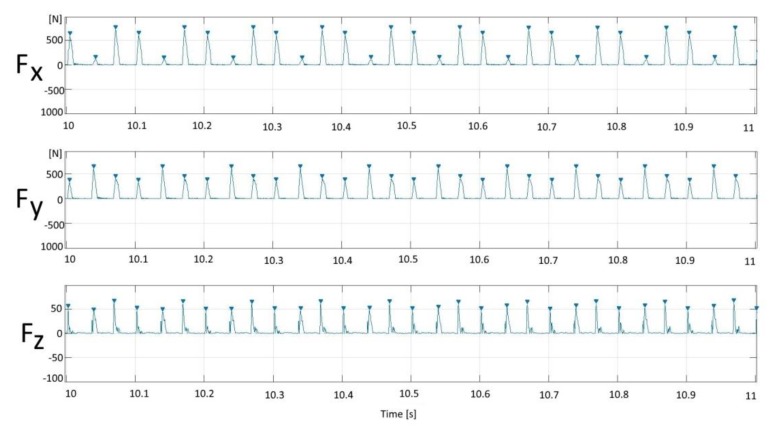
Typical dynamometer force readings.

The cutting tool was provided with three teeth. The dynamometer registers a peak for Fx, Fy, Fz, and Mz, respectively, every time a tooth enters the workpiece. Figure 5 shows a typical dynamometer reading. A program was written in MATLAB to find the peak value for each tooth entrance. The average peak value for each tooth was calculated separately, and their RMS value was calculated to get the feed force (Fx) and cutting force (Fy) shown in Figure 5.

Profilometry was performed with a Mitutoyo surface roughness-measuring instrument, Model SJ210 (Kanagawa, Japan). The measurement length was approximately 8 mm. White light interferometry with Zygo NewView™ 7300 (Middlefield, CT, US) was used to study the machined surfaces of the samples. Three square areas measuring 1 × 1 mm were analyzed for each sample. 

**Figure 6:**
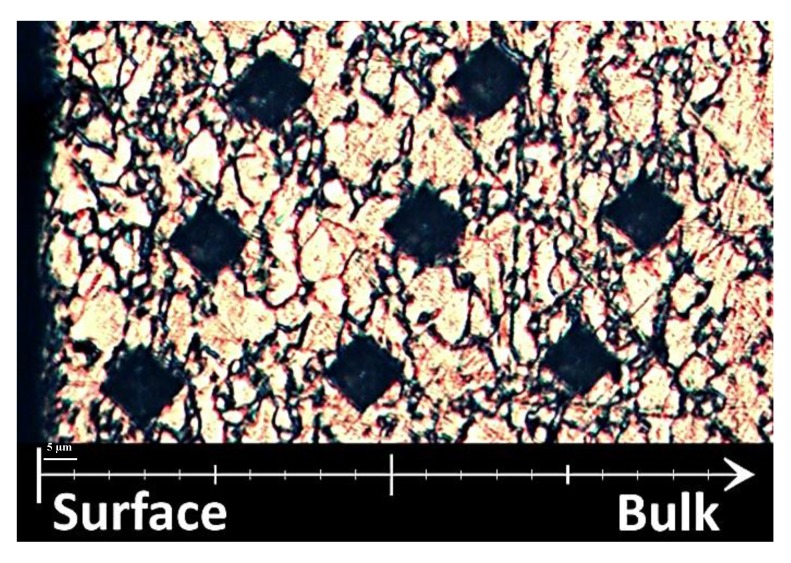
Example of microhardness indentations. The machined surface is on the left side and the indentations are perpendicular to this surface.

The microhardness test was performed using a KB Prüftechnik, model KB30s (Hochdorf-Assenheim, Germany) with indentations of HV 0.01 and an average of 3 indentations. The size of the indentations ranged between 6 and 10 µm in diameter. Figure 6 illustrates an example of a microhardness test.

Residual stress measurements were done with the Bruker D8Discover (Billerica, MA, US) with Co-Xrays using sine^2^Ψ method. Corundum powder was used for calibration. Tilting was performed along the cutting direction with an assumption of biaxial stress state. Young modulus was assumed to be 110 GPa and Poisson’s ratio 0.36.

## 3. Results and Discussion

### 3.1. Characterization of Workpiece Materials

Three different types of Ti-6Al-4V AM materials were investigated and compared against a forged reference sample. The chemical specifications of each material are shown in Table 4, and the mechanical properties of the forged titanium are shown in Table 5.

The AM materials were built-up either by laser metal deposition (LMD) or direct energy deposition (DED) technique with a laser source or by the powder bed fusion (PBF) technique with an electron beam (EB) source. The DED materials were fed either by wire or blown powder, as seen in Figure 1. The forged Ti-6Al-4V was hot rolled and mill annealed. The particle size and wire diameter for the selected materials in this study are shown in Table 6. Note that values presented in Table 4, Table 5 and Table 6 are from material certificates provided by aerospace suppliers or manufacturers.

The Ti-6Al-4V can adopt different microstructures which can be affected by the prior thermal and mechanical processes involved. The combination of process parameters will have a significant effect on the final microstructure of AM materials. The optimum microstructure required depends on the later application of the parts. For example, a martensitic material can be desired if a high strength material is required [19]. 

The samples from each AM method were cut and polished along two different directions and the microstructures were analyzed.

#### 3.1.1. Reference Material (Ref)

**Figure 7:**
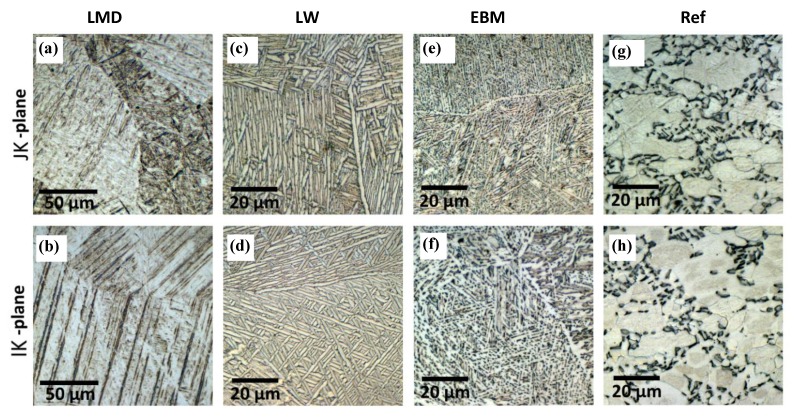
Microstructures of the three AM Ti-6Al-4V alloys and the forged reference. Images of LMD, LW, EBM, and reference materials were taken in the JK- (**a**,**c**,**e**,**g**) and IK-planes (**b**,**d**,**f**,**h**). Note that (**a**,**c**) are perpendicular to the build direction, and (**b**,**d**,**e**,**f**) are within the built plane.

The typical microstructure of Ti is a fully α microstructure. Alloying elements, such as aluminum and vanadium (α and β stabilizers, respectively), are usually added which results in α + β alloys. In consequence, the common microstructure of Ti-6Al-4V is an equiaxed microstructure with α and β phases. The β phase typically starts at the grain boundaries [36]. The α + β microstructure is typically formed during the recrystallization that follows slow cooling. Such microstructures are characterized by high ductility (and high fatigue resistance) and low hardness hence the need for forging and strain hardening. Our reference material is shown in Figure 7g,h and presented a mixed α + β equiaxed phase structure. The grain sizes were in the range of 10 µm.

#### 3.1.2. Laser Metal Deposition (LMD)

The microstructure of LMD-fabricated Ti-6Al-4V components is composed of acicular α’ martensite. Figure 7a,b shows such a microstructure as was also described in other studies [14,15,16,17,20]. The α’ martensitic microstructure produced after LMD manufacturing of Ti-6AL-4V is located inside the elongated prior β grains. These grains grow in an epitaxial manner during the layering deposition process. Due to the epitaxial nature of the build, the prior β grains will grow parallel to the build direction. This causes a columnar grain growth, related to the previous layer, in the direction of the highest temperature gradient [37].

The martensitic α’ structures have thin interfaces characterized by high dislocation densities between laths. Unlike α structures, these high dislocation densities can play the role of a barrier against dislocation movement under high mechanical strain, such as during machining. Also important to mention is the typical high hardness and strength of a martensitic structure compared to a cast or forged Ti-6Al-4V as will be shown in the later sections.

The grain size of the prior β grains is typically related to the time during which the temperature is above the β-transus temperature. For Ti-6Al-4V, this temperature is approximately 995 °C [38].

#### 3.1.3. Direct Energy Deposition by Laser Wire (LW)

The LW DED material shows a clear basket weave, Widmanstätten type of microstructure (Figure 7c,d). With AM, the metastable fine acicular martensite is associated to the embedded energy density. After the reheat cycle from the following build, a Widmanstätten structure can form from the previous fine acicular martensite. This is accompanied by a coarsening of the lamellar α phase. With an increase in bulk energy density, α lamellae can grow coarser [39,40,41].

We should note that α’ martensite is the consequence of diffusionless processes, in contrast with an α + β Widmanstätten structure which is created by a diffusion process [42].

#### 3.1.4. Electron Beam Melting (EBM)

Previous work on EBM Ti-6Al-4V showed that each melt pass increases the average width of α phase lenticular grains from 3 µm up to 6 µm [43]. It was noticed that the increase in width was accompanied by a decrease in hardness. As the built volume increases, the cooling rate increases—due to the higher heat transfer with a larger built-up volume—and the width of the lenticular α grains decreases [43]. Figure 7e,f shows such a case with the resulting α phase acicular formation. Similar work also found that prominent acicular α plates resulted from an EBM process [44]. Therefore, the different microstructures resulting from the EBM and LMD processes are mainly attributable to the differences in their cooling rates. This will, in turn, control how the β phase transforms to an α phase during the cooling cycle after the β-transus temperature is reached. If the cooling rate is high enough (approximately 10^6^ K/s), the α phase can transform into α’ martensite after the laser heating [17].

### 3.2. Machined Surface Hardness

The surface hardness of the AM materials was tested by indentation. Table 7 shows the samples’ hardness. All the AM materials proved to be harder than the reference material. The hardest material was the LMD, followed by the LW and the EBM with equal hardness. It is commonly known that microhardness is linked to the evolution of the microstructure of the built Ti-6Al-4V alloy during repetitive heat cycles hence the effects on the measured hardness values.

The LMD sample, which is an α′ martensite material, showed to be 8% harder than the EBM material which was produced by EBM. As noted in another work [45], the use of the laser beam method for producing a Ti-6Al-4V material results in α′ martensite. Laser beam involves faster cooling than the EBM method and is consequently more prone to result in an α′ phase and a harder material.

### 3.3. Cutting Forces

**Figure 8:**
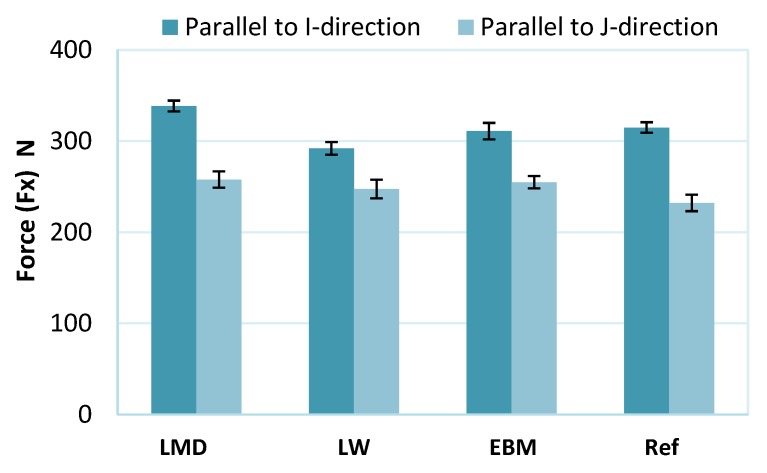
Forces along X-direction (feed) for the LMD, LW, EBM, and reference (Ref) materials.

The forces Fx along the feed direction X were higher for all materials when machining parallel to the I-direction than when machining in the J-direction, indicating material anisotropy (Figure 8). The reference material also showed differences in Fx when cut in different directions. Although its microstructure shared similarities to an equiaxed type, the reference material showed signs of anisotropy, as will be described later (Section 3.4).

**Figure 9:**
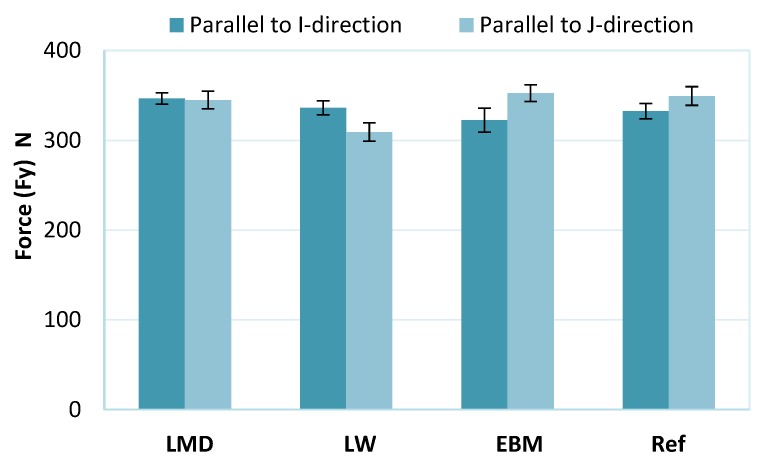
Forces perpendicular to the feed in the Y-direction for the LMD, LW, EBM, and reference (Ref) materials.

The forces perpendicular to the feed (Fy) in both machining directions were equal for the LMD material (Figure 9). For the LW material, Fy was higher when machining in the I-direction than in the J-direction; however, the opposite was observed for the EBM and reference materials. Note here that the Fy forces do not show significant differences.

Comparing the differences in the I- and J-directions, the force Fx along the feed was more affected than Fy by material anisotropy. The LW sample was the only material which exhibited both higher Fx and Fy forces in the I-direction. We must note that for all samples, the variations of Fy forces were not clearly beyond the margin of error; therefore, no clear trend could be observed for forces perpendicular to the feed direction.

It was observed when turning textured workpieces that due to the grain boundary effects, the main cutting force was much more affected than the thrust force [35]. In milling, since the tool engagement is very low (0.5 mm), a large portion of the resulting cutting force will be along the feed direction (along the X -axis) which can explain why the feed forces Fx were more affected than the forces along the Y -axis (Figure 8 and Figure 9). The X- and Y-directions are shown in Figure 3.

The LMD material, characterized by α’ martensite, had the highest force Fx along the feed in both the I- and J-directions with values comparable to the EBM material in the J-direction. This can be attributed to the hard martensite. Indeed, the LMD material had the highest hardness value (40 HV). 

### 3.4. Machined Surface and Strained Layer 

Conventional machining has been shown to alter the microstructure of workpiece materials [46]. Depending on the cutting conditions, wet milling of Ti-6Al-4V with cemented carbides was shown not to deform the surface material grains [8,47]. More recent work reported a highly strained area near the cut surface. Plastic strain was observed in the form of grain deformation after dry cutting of Ti-6Al-4V [11,48].

**Figure 10:**
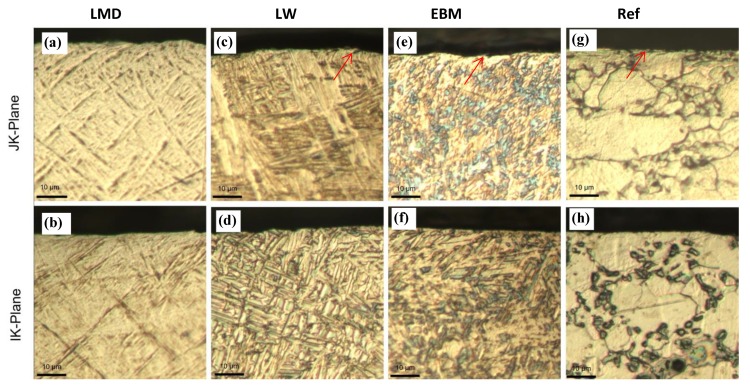
Machined layer and subsurface deformation of the three AM Ti-6Al-4V alloys and the forged reference. Images of LMD, LW, EBM, and reference materials were taken in the JK- (**a**,**c**,**e**,**g**) and IK-planes (**b**,**d**,**f**,**h**). Arrows indicate deformed grains. Note that (**a**,**c**) are perpendicular to the build direction and (**b**,**d**,**e**,**f**) are within the built plane.

Observation of the surface layer of the machined samples showed grain deformation at the near surface level (Figure 10). Careful microscopy observations of the grains revealed that the strained grains were confined to a subsurface level of less than 5 µm and slightly less for the LMD material. This can be attributed to the higher hardness of the LMD material.

### 3.5. Surface Profilometry 

**Figure 11:**
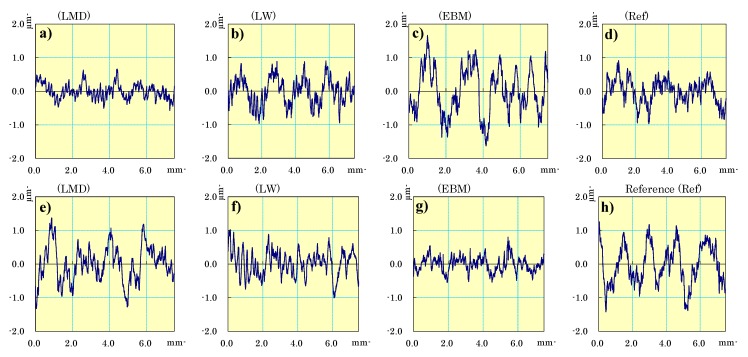
(**a**–**d**) Profilometry measurements conducted in the machining direction in the IJ-plane. The samples were machined in the I-direction. (**e**–**h**) Profilometry measurements conducted in the machining direction in the IJ-plane. The samples were machined in the J-direction.

The profilometry measurements were conducted in the machining direction in the IJ-plane. Measurements parallel to the I-direction (see Figure 4) are shown in Figure 11a–d, and measurements parallel to the J-direction (see Figure 4) are shown in Figure 11e–h.

**Figure 12:**
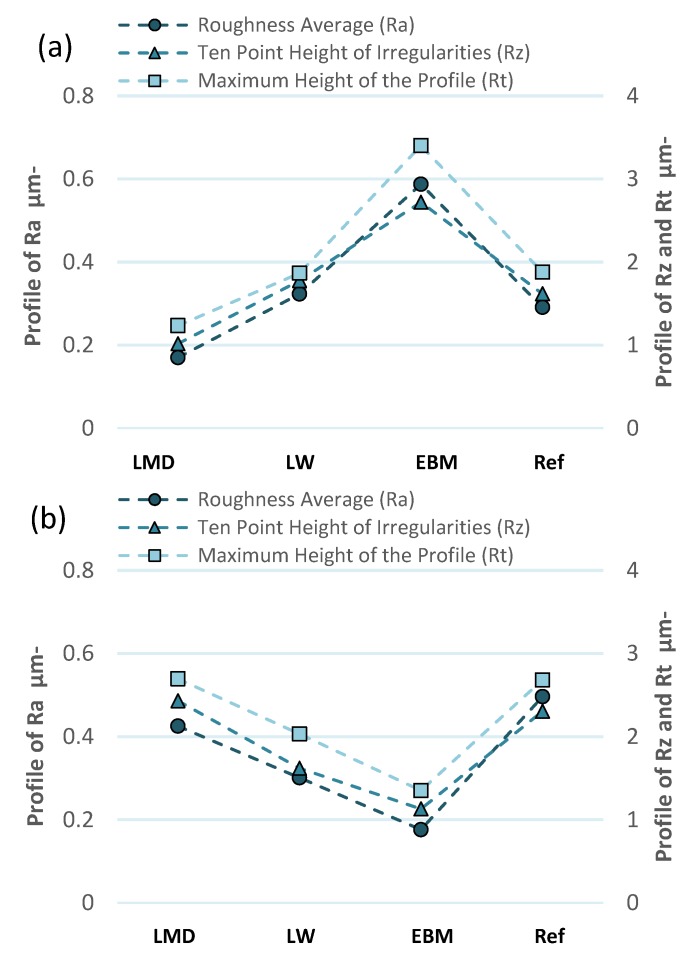
Summary of the profilometry measurements based on roughness average (Ra), ten-point height of irregularities (Rz) and maximum height of the profile (Rt). (**a**) Measurements conducted parallel to the machining direction in the IJ-plane. The samples were machined in the I-direction. (**b**) Measurements conducted parallel to the machining direction in the IJ-plane. The samples were machined in the J-direction.

The LMD material generated a smoother surface when machined along the I-direction than along the J-direction (Figure 12). The cutter was most affected by the microstructure in the JK-plane (Figure 7). The rougher surface finish, which was formed when machining parallel to the J-direction, may depend on the more numerous grain boundaries located in this direction of the martensitic microstructure. More grain boundaries result in a more hard-to-cut material which may generate a coarser surface. The LMD material is, by definition, built parallel to the I-direction. Consequently, it is easier to generate a smoother surface when machining the LMD material along the build direction.

The comparable surface roughness observed in both machining directions of the laser wire (LW) material can be attributed to the similar microstructures in the IK- and JK-planes in Figure 7. This means that the amount of grain boundaries in the α-Widmanstätten structure were in the same order of magnitude and generated a comparable surface roughness in the LW material when machined parallel or perpendicularly to the build direction.

The EBM material displayed a very coarse surface when machined along the I-direction and a lower surface roughness when machined along the J-direction. No clear differences in the microstructures in the IK- and JK-planes could explain the resulting effect on the surface roughness. It is worth noting that the build direction of the EBM material is parallel to the K-direction; however, the electron beam moved along the I-direction when it melted powder in a row to construct the solid workpiece. The beam switched rows along the J-direction and melted another row in the I-direction, like a raster pattern; however, the machining was performed only in the IJ-plane due to the part shape and a thinner dimension on the K axis.

When comparing the 3D printed samples with the forged reference, the LMD and LW materials had equal or finer surface finishes. However, compared to the reference material, the surface finish of the EBM material was coarser when machined parallel to the I-direction, but smoother when machined parallel to the J-direction.

### 3.6. Surface Topography Using White Light Interferometry

Since the grain sizes of our materials are in the range of a few microns, it is difficult to directly link the effect of microstructures on surface topography using profilometry. For a better analysis, surface measurements at a smaller scale were needed for relating any surface effect to the grains cut during machining. For that end, white light interferometry was performed on the machined surfaces.

**Figure 13:**
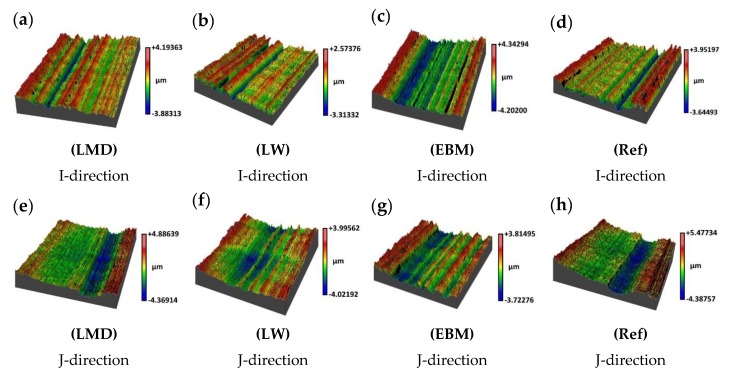
White light interferometry profile projections of analyzed 1 × 1 mm squares. One projection of each sample was selected amongst three measured and are referred to as (**a**–**h**). The feed direction is along the grooves, so from the bottom to the top of paper.

The results for the average surface roughness over a selected area in terms of arithmetical mean height (Sa), root mean square height (Sq), and peak–to–valley (PV) are shown in Table 8. Images of selected areas of the respective samples are displayed in Figure 13.

The main contribution to the surface finish was the cutter’s profile which is shown as trenches parallel to the machining direction. This means that the cutter’s profile has a greater influence on the surface roughness than the feed rate does.

Machining titanium alloys will start deteriorating the tool edge in the early stages of machining. Such small deteriorations will leave an imprint on the workpiece material and will appear as grooves. Although our measurements are in the same order of magnitude as typical grain sizes, the tool edge effect was the major contributor to the trenches seen in all samples.

### 3.7. Microhardness

**Figure 14:**
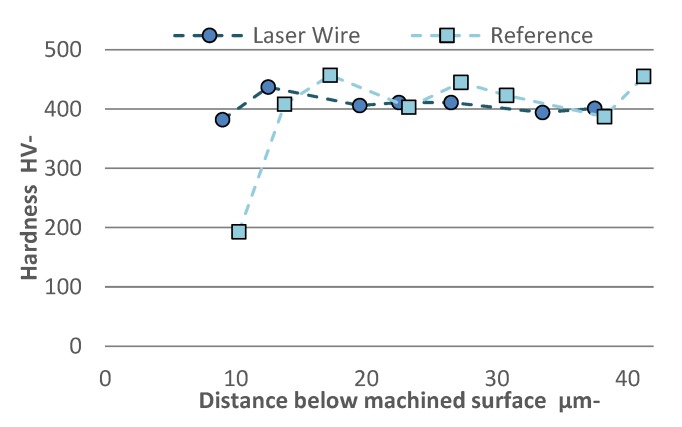
Microhardness profile of LW and reference materials. Measurements taken from the surface level into the bulk.

A microhardness indenter was used to measure the surface hardness as shown in Figure 6. The LW sample was used in comparison with the reference to assess if machining alters the surface hardness of AM materials. The LW material was a good candidate since it had the most compressive surface residual stress, as will be shown in the next section. The microhardness test indicates that both materials were softer near the machined surface (Figure 14). The penetration depth of the thermal softening induced by machining was of approximately 10 µm for the reference material. In the case of the laser wire material the influence of softening was much less pronounced at a similar depth. Interestingly, while the near surface hardness of the LW sample was higher than the reference, the hardness of both samples was comparable at deeper depths.

Strain hardening typically occurs at the machined surface. A similar procedure performed in a previous work showed an increase in hardness after machining at a level similarly close to the surface; however, the material was made from powder metal with a Ti-6Al-4V matrix [49]. Other previous work reported a softening effect when machining Ti-6Al-4V [50,51,52,53,54] which was explained by a phenomenon attributed to over-aging [50,51,52]. Other explanations were the transformation of the β phase near the surface in combination with the stress relaxation due to the cyclic temperature [53]. The nature of the edge effect was also reported, which may result in lower hardness values [54]; however, this work involved nano-hardness indentations performed very close to the surface (at approximately 1 µm). Therefore, there is no complete agreement of the surface softening effect. Over-aging is not applicable in the case of the present work, since the material used was not originally aged.

During cutting of titanium alloys, the temperature can exceed the recrystallization temperature of approximately 995 °C [38]. The cutting process involves a competitive action between the effects of thermal softening and strain hardening. Part of this heat will be transmitted to the material’s surface. Due to the low conductivity of titanium, the concentration of temperature will be confined to the near surface area and could promote a partial recovery. Therefore, if the material includes traces of dislocations, a partial recovery could explain the hardness reduction at the near surface area.

### 3.8. Residual Stress

An important parameter of surface integrity is residual stress. This is particularly important for manufacturers requiring a high fatigue life of parts such as in the aerospace sector. Residual stresses can develop due to the inherent heating and cooling cycles of AM. Applications for which surface cracks can occur during the parts’ lifetime also require careful monitoring of residual stresses. Tensile residual stresses have been correlated to crack growth and propagation [55].

Many previous studies reported the effect of cutting parameters and edge sharpness to the parts’ residual stress. An example is the cutting speed effect which does not have a linear relationship with the resulting stresses. Turning Ti-6Al-4V resulted in compressive stresses at speeds around 150 m/min or below; however, the stress became tensile above that speed [56].

**Figure 15:**
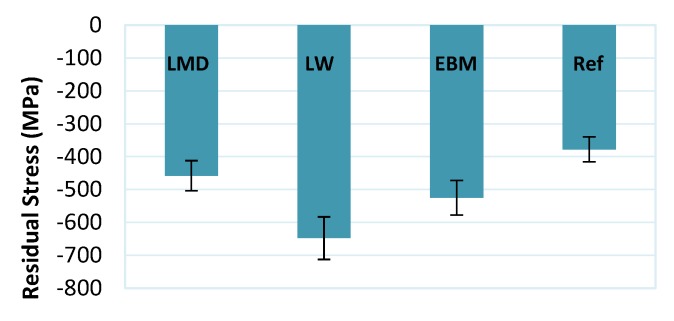
Residual stress measurements after machining.

Figure 15 shows the results of the residual stress, where all samples had compressive residual stresses. Compressive residual stresses can be beneficial for many applications where crack propagation can be a serious issue such as in the aerospace sector. The reference material clearly shows the least residual stress. This can be expected since the reference material, which was forged and mill annealed, was also highly affected by surface softening after machining, with the possibility of partial recovery as seen in the previous section. This, in turn, resulted in the smallest residual stress value. The LW material had the highest value and was much less affected by surface softening, followed by the PD and finally the LMD materials. 

We should note that the LMD material was a martensitic type of material which is typically associated with high dislocation densities generated during the diffusionless martensitic transformation [24]. This suggests that the strain associated with the machining action could not accommodate for additional dislocations as compared to other non-martensitic AM materials, and this could be a reason behind the lower stress values obtained with this material. 

### 3.9. Chip Geometry and Chip Formation

**Figure 16:**
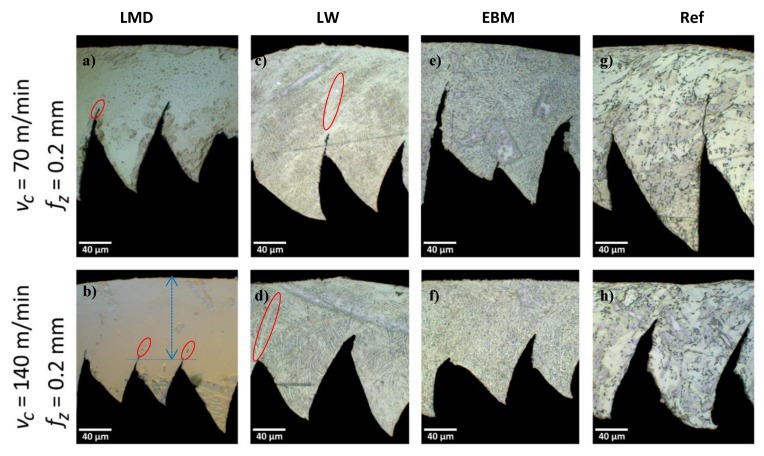
Chip geometry for LMD, LW, EBM, and Ref for two cutting speeds (*v_c_*) of 70 m/min (**a**,**c**,**e**,**g**) and 140 m/min (**b**,**d**,**f**,**h**). The feed rate (*f_z_*) was constant at 0.2 mm.

In order to identify if different AM techniques have different effects on machinability, multiple chip samples were polished and etched for analysis (Figure 16). Since the cutting speed has a direct relation to the chip segmentation frequency, two cutting speeds were chosen: a low speed of 70 m/min and a higher speed of 140 m/min. It is known that different materials produce different types of chips; therefore, a good indication that the materials’ properties are different is when they show differences in chip formation.

For practical reasons, the feed was increased to 0.2 mm/rev, as the 0.05 mm/rev feed produced chips too thin to allow handling and manipulations for polishing.

Comparing all chips in Figure 16, it is clear that the chip formation had dissimilarities indicating that the material properties are affected by the AM process. Observation of the chips from the reference material showed large clear segments which are typical of the segmentation type described in the literature for Ti-6Al-4V [49]. 

The LW sample produced clear adiabatic shear bands (ASB) (highlighted by red regions) with some signs of recrystallization inside [49] which can be seen as a brighter colored area. The LMD material produced the thickest chips when measured from the chip back to the first sign of segmentation (shown in blue). The α’ martensite can be responsible for the different chip formation. Close observation showed signs of cracks between two segments along the ASB (highlighted by red regions). Again, the brittle martensite could have prevented high strains in the vicinity of the ASB, resulting in cracks showing between segments.

The EBM material had typically smaller segment widths when compared to the reference sample. The difference in segmentation and chip formation is a clear sign of variations in the properties of materials manufactured by different AM processes.

### 3.10. Effect of Grain Texture Orientation on Machinability

Since different AM materials produce different microstructures, it is interesting to study the effect of grain texture orientation on machinability, especially on cutting forces. As indicated in the introduction, previous work [35] has shown that grain texture direction affects cutting forces when turning aluminum; however, no similar work has been done for milling. Since the process of metal removal in turning and milling have similarities, it would be fair to expect a similar effect in milling for materials with different texture orientations.

**Figure 17:**
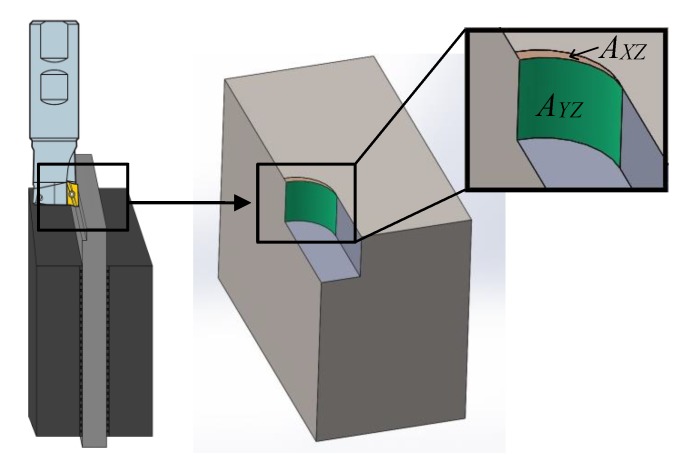
Machined volume showing the *A_YZ_* (green) and *A_XZ_* (yellow) areas.

Since the material is anisotropic, it is interesting to analyze the effect of cut areas when cutting along the I- or J-direction. The cut areas can be separated into two areas (Figure 17, in green and yellow). The yellow area is mainly affected by the feed, and the green area by the depth of cut, engagement, and tool diameter. The grains along the green area will be cut, whereas the ones inside the yellow area will form the chip.

The yellow area will be highly strained and form a segmented chip as is the case when cutting titanium alloys. Segmented chips are characterized by adiabatic shear bands (ASBs) where localized high strain, high strain rates, and high temperature occur [49].

The volume of work material that is removed each time the cutter hits the surface of the workpiece can be calculated with the formula in Equation (1).

(1) $$Removed\;volume\left(V_{R}\right)=f_{z}*a_{p}*a_{e}$$

where (*f_z_*) is the feed per tooth, (*a_p_*) is the axial depth of cut, and (*a_e_*) is the radial depth of cut.

The cutters’ (or teeth’s) impact on the workpiece must be calculated to compare the surface integrity against the microstructure of the work material. When machining along the X-direction or the feed direction (see Figure 3), the tooth enters the workpiece perpendicularly to the XY-plane (or IJ-plane in Figure 4) and leaves the workpiece perpendicularly to the YZ-plane (or JK in Figure 4). The tooth affects the YZ-plane (or JK in Figure 4) from where it enters until it leaves the workpiece and forms an arc between these points. The axial depth of cut (*a_p_*) affected by the tooth, times the arc length, represents the affected area in the YZ-plane (*A_YZ_*) and is calculated with Equation (2), where *r* is the radius of the cutting diameter of the tool.

(2) $$A_{YZ}=a_{p}*r*\cos^{-1}\left(\frac{r-a_{e}}{r}\right)$$

The affected area in the XZ-plane (*A_XZ_*) depends on the feed rate (*f_z_*) and the radial depth of cut (*a_e_*) and is calculated with Equation (3).

(3) $$A_{XZ}=f_{z}*a_{e}$$

The ratio between the affected areas in the YZ-plane and XZ-plane is a measure of the difference between the affected areas. This ratio is calculated according to Equation (4).

(4) $$R:Ratio\left(\frac{A_{YZ}}{A_{XZ}}\right)=\frac{a_{p}*r*\cos^{-1}\left(\frac{r-a_{e}}{r}\right)}{f_{z}*a_{e}}$$

In this study, *A_YZ_* is 569 times larger than *A_XZ_* when machining parallel to the X-direction compared to when machining parallel to the Y-direction.

To summarize, the green area is related to the number of grain boundaries cut depending on the texture direction which can affect cutting forces. The yellow area, which will form the chips and segmentation, is a region characterized by a high deformation of grains and grain boundaries. The smaller the yellow area is (for example, due to a reduction of feed), the higher the effect of grain boundaries will be [35], which in turn will increase the *R* ratio. This means that the calculated ratio *R* can be considered as a sensitivity indicator of the effect of the texture/cut direction on cutting forces. In other words, the higher the *R* value, the higher the effect of texture on cutting forces.

Previous work done on micro-cutting textured Al related the cutting forces to the material texture and grain boundary [35]. It was found that the workpiece texture had a high influence on cutting forces for small cuts or micro cuts. It was difficult to relate exact texture directions to cutting forces, but it was observed that the cutting force varied several times along the grain boundaries. Following this, we will focus on the micro cut region during the milling process in the next section, where the material texture will have a larger influence on cutting forces and therefore on surface integrity.

This study does not intend to be a comprehensive texture to machining research work. This is beyond the scope of this paper and should rather be addressed in a separate work due to the fact of its complexity. However, the intention was to pinpoint how the machining direction can interact with the material texture. 

During up-milling, the maximum thickness of the chip will be produced at the beginning of the cutting process and will go down towards 0 at the end of the cut. Since our feed was 50 µm, a portion of the chip thickness will be below 10 µm, which is in the range of our microstructures. As noted in Section 3.3., it is worth to keep in mind that when the tool engagement is very low, a large portion of the resulting cutting force will be along the feed direction (along the X-axis).

**Figure 18:**
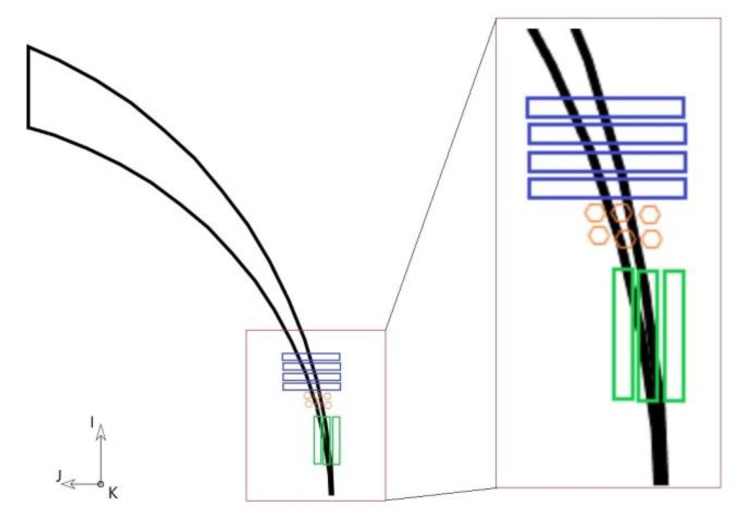
Areas cut in each cycle with green grains along the cut (I-direction), blue grains perpendicular to the cut (J-direction), and orange grains perpendicular to the figure plane (K-direction). This figure represents the top side of the workpiece shown in Figure 17.

By analyzing the cut region where the chip thickness is in the range of our microstructures, we can identify three cases (Figure 18, zoomed-in part). If the material texture is along the I-direction (case 1, Figure 18 in blue), more grain boundaries will be cut compared to if the grains are along the J-direction (case 2, Figure 18 in green). If the texture is along the K-direction (case 3, Figure 18 in orange), fewer grains boundaries will be cut, as they are mainly removed inside the yellow area in Figure 17.

This shows how machining can be affected by the amount of cut grain boundaries and depending on the direction of cut. The three identified cases can contribute to different cutting forces depending on the cutting direction.

In the case of our AM materials, the texture did not seem to follow a specific I-, J- or K-direction, making it difficult to directly correlate it with measured machining variables such as forces (as mentioned also in previous work [35]). However, the relation of texture direction to machining direction will still play a role and affect machinability as was also described in Reference [35].

Using the above, one can relate the effect of the cutting parameters to the texture direction. A practical example is when finishing a product with minimal material removal, where a small feed and tool engagement will be used. In that case, the previous calculated ratio *R* (Equation (4)) will be even larger, due to the smaller yellow area. To be specific, a larger portion of the cut will be in the micro cut region. Under such conditions, the cutting forces should be more sensitive to the texture direction with an increase in the value of *R*.

## 4. Conclusions

Additive manufacturing applications have been growing in many industries such as in the aerospace and automotive sectors. Many parts requiring tight tolerances and specific surface properties still require a subsequent subtractive manufacturing process such as machining.

This work was not intended to propose the best AM method, since different parts made by manufacturers require different properties and surface qualities depending on their application. Each AM method has its own parameters which affect the material; however, we should be aware that the SM will further alter the material’s surface properties depending on the cutting parameters.

This research compares the surface integrity of Ti-6Al-4V parts made by three different AM processes combined with SM. Each AM process resulted in completely different material microstructures. The LMD material had an α’ martensitic structure, the laser wire (LW) material a Widmanstätten structure and the EBM powder bed (EBM) material showed an α phase acicular formation. The conventional forged reference displayed an equiaxed α + β phase.

All the samples were finish machined in two different directions by milling and the cutting forces were evaluated. The forces showed differences mainly in the feed direction due to the material anisotropy; however, the forces perpendicular to the feed direction were less affected by the microstructures of the different materials.

Differences were also observed depending on which type of AM material was machined. The highest feed force was for the LMD due to the fact of its hard martensite structure. The LMD also showed the largest difference in feed force between the two different machining directions due to the fact of its high anisotropy.

The surface integrity was analyzed after machining and the depth of the strained material was measured. All samples showed a strained surface at nearly equivalent depth, except the LMD material which had the lowest subsurface level strain. This can also be attributed to its hard martensitic structure.

The subsurface hardness was also measured to verify if AM materials can behave differently when compared to the reference material under the high stress, strain, and temperature resulting from the machining process. It was observed that the near surface layer of the AM material was less affected and less softened than that of the reference material. The softening effect was explained by a possible partial recovery due to the concentration of high temperature at the near surface area while machining.

To help understand the combination of microstructure texture and machining direction on cutting forces, a micro level analysis was performed, and a relation was made between the milling action and the amount of grain boundaries intercepted during cutting. The analysis showed that the effects of texture orientation can be related to cutting forces, especially when finish machining with a very small chip area. This was also shown by an increase in the sensitivity ratio *R*.

Surface roughness was analyzed for each sample. The LMD and EBM materials had the highest divergence in roughness between the different machining directions due to the material anisotropy.

Chip segmentations resulting from the SM process were analyzed. Typically, different materials produce different chips. Therefore, the chip formation is a good indicator of differences in material properties. In fact, each AM sample produced different chips. The LMD material had the thickest chips, while the LW showed clear ASB with changes in the microstructure inside the bands. The reference showed large segments typical for Ti alloys.

Residual stress analysis was performed by XRD after machining. All the samples showed compressive residual stresses which is desired for many applications. The reference material had the least residual stress. This was explained by the possible partial recovery effect due to the localized increase in temperature subsequent to machining.

It is common nowadays to find numerous AM parts in industries. Many AM parts are later machined before they are finally used in assemblies. For some end users, surface integrity is a very important parameter to consider before moving towards a practical application. It can be a challenge to predict the surface integrity of parts since adding an SM process to AM parts can increase the complexity of the changes occurring at the surface. This research helps understanding how the surface integrity is affected when different AM processes are combined to SM.

## Figures and Tables

**Table 1:** Tool specifications.

Tool	R390-016A16-07M
Cutting diameter mm	16
Tool overhang length mm	32
Number of teeth	3
Inserts	390R-070208M-MM S30T
Coating	PVD (Ti, Al) N

**Table 2:** Milling operation parameters.

**Cutting Speed, *v_c_***	70 m/min
**Feed Rate, *f_z_***	0.05 mm/tooth
**Axial Depth of Cut, *a_p_***	5 mm
**Radial Depth of Cut, *a_e_***	0.5 mm

**Table 3:** Build direction.

Sample	Build Plane Deposition Parallel to *
Laser metal deposition (LMD)	I-direction
Laser wire (LW)	I-direction
Electron beam melting (EBM)	K-direction

* I-, J-, and K-directions as shown in Figure 4.

**Table 4:** Chemical specifications of the workpiece materials. Data are in weight %.

	LMD	LW	EBM	Reference Material
Aluminum	6.23	6.04	6	6.47
Vanadium	4.21	3.95	4	4.07
Carbon	0.01	0.02	0.03	0.14
Iron	0.18	0.13	0.1	0.15
Oxygen	0.11	0.15	0.15	0.17
Nitrogen	0.007	0.01	0.01	0.003
Hydrogen	0.002	0.002	0.003	0.001
Titanium	Balance	Balance	Balance	Balance

**Table 5:** Mechanical properties of the reference (Ref) Ti-6Al-4V.

Mechanical Properties	Longitudinal	Transverse
Proof stress, R_p0.2_ MPa	921	928
Tensile strength, MPa	975	987
Elongation 50 mm, %	16.5	16.5
Hardness, HV	350	-

**Table 6:** Powder particle size and wire diameter of the AM raw materials.

	Particle Size	Wire Diameter
LMD	45–75 µm	-
LW	-	1.143 mm
EBM	45–100 µm	-

**Table 7:** Average hardness of AM and reference materials based on three measurements.

	Hardness (HRC)
LMD	41
LW	38
EBM	38
Reference	36

**Table 8:** Data of 3D surface roughness parameters: arithmetical mean height (Sa), root mean square height (Sq), and peak-to-valley (PV). These data are shown parallel to the I-direction and parallel to the J-direction for all samples.

Sample	Sa µm	Sq µm	PV µm
LMD parallel to I	0.87 ± 0.09	1.07 ± 0.09	7.81 ± 0.19
LMD parallel to J	0.94 ± 0.05	1.20 ± 0.04	8.74 ± 0.37
LW parallel to I	0.69 ± 0.15	0.85 ± 0.15	7.96 ± 2.14
LW parallel to J	0.95 ± 0.03	1.15 ± 0.02	7.36 ± 0.80
EBM parallel to I	0.93 ± 0.15	1.12 ±0.15	7.80 ± 0.55
EBM parallel to J	1.33 ± 0.05	1.58 ± 0.05	8.59 ± 0.68
Ref parallel to I	0.93 ± 0.04	1.15 ± 0.05	7.62 ± 0.09
Ref parallel to J	1.04 ± 0.25	1.27 ± 0.28	8.21 ± 1.35
